# Factors Associated with University Students’ Deterioration from Subthreshold Depression to Depression before and during the COVID-19 Pandemic

**DOI:** 10.3390/bs13010072

**Published:** 2023-01-13

**Authors:** Koki Takagaki, Satoshi Yokoyama

**Affiliations:** 1 Health Service Center, Hiroshima University, Higashi-Hiroshima 739-8514, Japan; 2 Department of Psychiatry and Neurosciences, Hiroshima University, Hiroshima 734-8551, Japan

**Keywords:** subthreshold depression, major depressive disorder, behavioral activation, avoidance behavior, self-compassion

## Abstract

COVID-19 has exposed university students to high-stress situations, and the percentage of individuals with depressive symptoms was high during the COVID-19 pandemic. Furthermore, subthreshold depression carries a risk for the subsequent development of major depressive disorder (MDD). During the COVID-19 pandemic, we examined whether differences exist between university students who deteriorated from subthreshold depression to MDD and those who remained stable or improved. Four hundred seventeen participants completed all the measures twice over a one-year interval. One hundred twenty-three participants met the criteria for subthreshold depression at Time 1. One year later, 42 participants no longer met the criteria for subthreshold depression, 68 participants maintained the criteria for subthreshold depression, and 13 participants met the criteria for MDD. We conducted two-way repeated measures ANOVA to examine the differences between those who deteriorated from subthreshold depression to MDD and those who did not. The study results suggest that avoidance behavior is associated with the development of MDD from subthreshold depression. Additionally, the study showed that experiencing isolation relates to MDD onset. Therefore, we should monitor avoidance behavior and isolation in pandemic conditions. Consequently, attention to avoidance behavior and isolation may be important; however, further research is required.

## 1. Introduction

University students have been exposed to high-stress situations due to COVID-19, and consequently mental health effects have been reported [1]. A survey of the percentage of people with depressive symptoms in various populations, including university students, health workers, infected patients, and the general public, found that university students had the highest prevalence at 43.3% [2]. A study in Japan reported that young people (ages 18–29) have a higher percentage of depressive symptoms [3]. Therefore, investigating depressive symptoms among university students during the COVID-19 pandemic is essential.

Subthreshold depression includes clinically significant depressive symptoms that do not meet the diagnostic criteria for Major Depressive Disorder (MDD [4,5]). A previous study suggested that the estimated prevalence of subthreshold depression is higher than that observed for MDD, with evidence that up to 29.2% of adolescents are affected by it [6]. People with subthreshold depression face an elevated risk for subsequent development of MDD in the future [7,8]. Moreover, another study reported that people with subthreshold depression were approximately twice more likely than non-depressed people to develop MDD [9]. Subthreshold depression can engender severe functional impairment, adversely affecting academic performance and social activity [10]. Furthermore, subthreshold depression was associated with an increased lifetime prevalence of suicide attempts [11]. A recent review article suggested that subthreshold depression in adolescence is clinically relevant [6]. Therefore, it should be targeted for early intervention. However, the meta-analysis failed to detect significant effects on the incidence of MDD at follow-up between psychological intervention and control groups for subthreshold depression [12]. Clarifying factors in subthreshold depression’s progression to MDD would provide a fundamental perspective supporting more effective treatments for university students with subthreshold depression during unusual times, such as the COVID-19 pandemic.

Behavioral activation is one of the effective psychotherapies for treating subthreshold depression. The one goal of behavioral activation is to increase behavior based on one’s values and restore an environment characterized by diverse and stable sources of positive reinforcement [13,14]. Additionally, behavioral activation places emphasis on avoidant behaviors [14]. Avoidant behaviors function to alleviate individuals’ distress in the short term, but can also increase depressive symptoms in the long term [14]. One of the key factors in avoidant behavior change are values. Values are defined as freely chosen, verbally constructed consequences of ongoing, dynamic, evolving patterns of activity, which establish predominant reinforcers for that activity that are intrinsic in the engagement in the valued behavioral pattern itself [15]. Behavioral activation suggests that a behavior might become automatically reinforced simply by association with long-term valued consequences, and this association might sustain behavior in the face of extremely strong competing short-term contingencies [16]. Therefore, in the context of behavioral activation, the main components are activation, avoidance, positive reinforcement, and value-based behaviors [17]. In other words, behavioral activation is used to modify a person’s environment through behavior change, which in turn increases access to positively reinforcing events and activities based on their own values [13]. The effectiveness of behavioral activation has been shown for university students with subthreshold depression [18,19,20]. A previous meta-analysis reported that behavioral activation for subthreshold depression significantly affected depression in the short term, but had no significant effect at one-year follow-up [21]. Another study reported that behavioral activation is more effective for university students with subthreshold depression compared to a control group; however, no significant difference in MDD incidence was detected at one-year follow-up [22]. Although prevention interventions are critical for people with subthreshold depression [9], few articles have shown long-term effects using behavioral activation in university students with subthreshold depression. It is noted that there is still a lack of research on mechanisms related to behavioral activation [23]. Further research is needed to determine whether these factors are associated with the transition from subthreshold depression to depression in the context of behavioral activation.

Research has been conducted on the relationship between behavioral activation and self-compassion. Acting according to one’s values is stressful and challenging [24]. Nevertheless, self-compassion may promote value-based behaviors when confronting obstacles (e.g., [25]). In addition, self-compassion was positively correlated with activation [26], while self-compassion and avoidance were inversely correlated [26,27]. Self-compassion comprises six components: self-kindness, common humanity, mindfulness, self-judgment, isolation, and over-identification [28]. Self-kindness, common humanity, and mindfulness are related to value-based behaviors [29]. Self-compassion has not been considered a main component in behavioral activation. However, research on behavioral activation and self-compassion has recently gained momentum, and self-compassion might be an important component of behavioral activation. Therefore, when considering whether it is related to the change from subthreshold depression to depression in the context of behavioral activation, including self-compassion might provide new insights. Although early intervention for treating subthreshold depression in adolescents is critical for addressing barriers to treatment response and remission, the efficacy of treatment in adolescents with subthreshold depression is understudied [6]. Therefore, the aim of this study was to examine whether the components of behavioral activation and self-compassion are related to the deterioration of university students’ mental health, from subthreshold depression to MDD.

## 2. Materials and Methods

### 2.1. Participants and Procedures

In this study, undergraduate students living in Japan were recruited by simple random sampling as participants from Rakuten Insight Inc. (Tokyo, Japan), a market research company that conducts online surveys. The data were collected via the Internet in 2019 (Time 1; T1: pre-COVID-19 pandemic) and 2020 (Time 2; T2: during the COVID-19 pandemic). First, an online questionnaire was sent to the participants from Rakuten Insight Inc. Next, the participants were asked to read an explanation of the study and to provide informed consent by clicking on the button signifying agreement, before responding to the questionnaire. Only those who voluntarily agreed to participate in the study responded to the questionnaire. At T1, Rakuten Insight Inc. recruited 800 university students with a 1:1 male-to-female ratio as study participants. As a result, 385 male and 415 female university students participated in the study. One year after T1, university students who had participated in the questionnaire survey in T1 were contacted via Rakuten Insight Inc. and invited to participate again in the study. In total, 417 participants (52.13%) completed all the measures at T1 and T2 (244 women, 173 men; mean age (T2) = 21.54 yr.; SD = 1.42). The study protocol was reviewed and approved by the Ethics Committee of Hiroshima University (approval number: E2019-1644). All procedures performed in the study were conducted following relevant ethical standards.

### 2.2. Measures

#### 2.2.1. Japanese Version of the Center for Epidemiologic Studies Depression Scale (CES-D [30])

Radloff [31] developed the original Center for Epidemiologic Studies Depression Scale (CES-D) and demonstrated its reliability and validity. The original CES-D comprises 20 items that are scored on a four-point scale (1: rarely or never (less than one day), to 4: most or all the time (5–7 days) [31]. The Japanese CES-D has good reliability and validity [30] for measuring depressive symptoms.

#### 2.2.2. Japanese Version of the Patient Health Questionnaire (PHQ-9 [32])

The original version of the Patient Health Questionnaire (PHQ-9) consists of nine items rated on a four-point Likert scale ranging from 0 (not at all) to 3 (nearly every day) [33]. The Japanese PHQ-9 has good reliability and validity [32]. However, due to ethical considerations, the survey was conducted with eight items only. An item related to suicide was deleted following Rakuten Insight’s Compliance Guidelines. Furthermore, in Internet surveys, it is possible to remove the item related to suicide from the PHQ-9 without affecting its reliability and validity [34].

#### 2.2.3. Japanese Version of the Behavioral Activation for Depression Scale (BADS [35])

Kanter et al. [36] developed the original Behavioral Activation for Depression Scale (BADS), demonstrating its good reliability and validity. The original BADS comprises four subscales and 25 items rated on a seven-point Likert-type scale (0: not at all, to 6: completely) [36]. Its four subscales include Activation (BADS-AC), Avoidance/Rumination (BADS-AR), Work/School Impairment (BADS-WS), and Social Impairment (BADS-SI). The BADS-AC measures goal-directed activation and the completion of scheduled activities. The BADS-AR measures avoidance of a negative aversive state and engagement in rumination, rather than active problem solving. Takagaki et al. [35] developed and demonstrated the reliability and validity of the Japanese version of the BADS. In our study, we used only BADS-AC and BADS-AR to examine the factors related to changes in clinical condition one year later.

#### 2.2.4. Japanese Version of the Valuing Questionnaire (VQ [37])

Smout et al. [38] developed the original Valuing Questionnaire (VQ), demonstrating its reliability and validity. The VQ comprises two subscales and 10 items rated on a five-point Likert-type scale (0: not at all, to 6: extremely) [38]. Its two subscales include the Valuing Questionnaire—Progress (VQ-Progress) and Valuing Questionnaire—Obstruction (VQ-Obstruction). Here, VQ-Progress reflects the enactment of values, including clear awareness of what is personally meaningful and perseverance. Consequently, VQ-Progress reflects value-based behavior. On the contrary, VQ-Obstruction reflects the disruption of valued living because of avoidance of unwanted experiences and distraction from values by inattention to values or attention to other psychological experiences. Doi et al. [37] developed and demonstrated the reliability and validity of the Japanese version of the VQ that was used in this study.

#### 2.2.5. Japanese Version of the Environmental Reward Observation Scale (EROS [39])

Armento and Hopko [40] developed the original Environmental Reward Observation Scale (EROS) and demonstrated its reliability and validity. The original EROS comprises 10 items scored on a four-point Likert-type scale (1: strongly disagree, to 4: strongly agree) [40]. The Japanese version of EROS has good reliability and validity [39]. It measures exposure to environmental rewards deemed necessary for increasing response-contingent positive reinforcement.

#### 2.2.6. Japanese Version of the Brief Version of the Self-Compassion Scale (SCS–SF [41])

Raes et al. [42] developed the original brief version of the Self-Compassion Scale (SCS–SF), demonstrating its reliability and validity. The original SCS–SF comprises four subscales and 12 items rated on a five-point Likert-type scale (1: almost never, to 5: almost always) [42]. The scale’s six subscales include self-kindness, common humanity, mindfulness, self-judgment, isolation, and over-identification. The Japanese version of the SCS–SF has good reliability and validity [41]. Therefore, we used all six subscales in this study to examine the factors related to changes in clinical condition one year later.

### 2.3. Definitions of Participating Groups

We defined the depressed, subthreshold depression, and healthy groups based on the following criteria. The depressed group comprised participants who always included an item from the PHQ-9 based on the DSM-IV criteria, for example, “Little interest or pleasure in doing things” or “Feeling down, depressed, or hopeless”, answered more than five of the PHQ-9 items on more than half of the days, and had a CES-D score of at least 16. The subthreshold depression group included those who did not meet the DSM-IV criteria for MDD using the PHQ-9 described above and had a CES-D score of 16 or higher. Finally, the healthy group included those who did not meet the DSM-IV criteria for MDD and subthreshold depression. At one-year follow-up, the participants who fit into the healthy group from the subthreshold depression group were defined as the subthreshold depression-improved group, those who still fit into the subthreshold depression group were defined as the subthreshold depression-maintained group, and those who fit into the depression group from the subthreshold depression group were defined as the subthreshold depression-worsened group.

### 2.4. Statistical Analyses

In this study, we examined whether differences exist between those who did and did not progress from subthreshold depression to MDD during the COVID-19 pandemic. Participants falling into the subthreshold depression group at T1 (before the pandemic) were included in the analyses. First, we reported the descriptive data. Demographic and clinical characteristics (sex; Are you a member of any club or circle activities in the university?; Have you ever consulted health service center or student counseling service of the university for mental health problems?; Have you ever visited a hospital (psychiatry or psychosomatic medicine) for mental health problems?) were investigated using the Fisher’s exact test. Additionally, age, CES-D (T1), and CES-D (T2) were investigated using one-way ANOVAs. Two-way repeated measures ANOVAs were conducted for the behavioral characteristics and self-compassion data, to examine the differences among the three groups (subthreshold depression-improved group, subthreshold depression-maintained group, and subthreshold depression-worsened group). For our analyses, we used statistical software (SPSS Version-28; SPSS Japan Inc., Tokyo, Japan).

## 3. Results

### 3.1. Participants and Descriptive Statistics

In T1, participants were allocated to the healthy (*n* = 260), subthreshold depression (*n* = 123), and depressed groups (*n* = 34) based on CES-D and PHQ-9. The results showed that 29.50% of university students met the criteria for subthreshold depression. Next, we examined the progress of the subthreshold depression group one year later. Forty-two participants had progressed from subthreshold depression to healthy (subthreshold depression-improved group), 68 maintained their levels in the subthreshold depression group (subthreshold depression-maintained group), and 13 participants deteriorated from subthreshold levels to depression (subthreshold depression-worsened group) one year later. The results showed that during the COVID-19 pandemic, 10.57% of university students deteriorated from subthreshold depression to MDD. In addition, 34.15% of university students no longer met the criteria for subthreshold depression. Table 1 shows the demographic information for each group.

### 3.2. Two-Way Repeated Measures ANOVA Results

**Figure 1:**
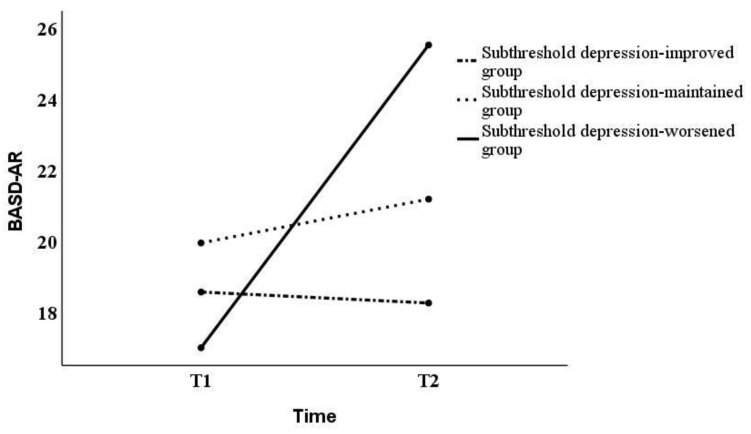
Changes in BADS-AR score from T1 to T2. BADS-AR, Behavioral Activation for Depression Scale—Avoidance/Rumination.

**Figure 2:**
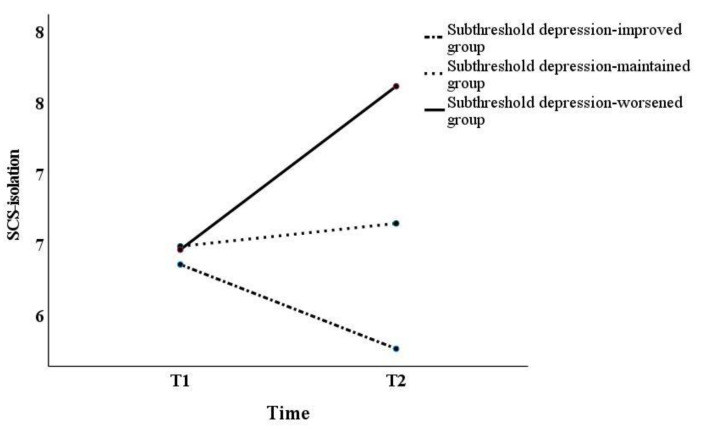
Changes in SCS-Isolation score from T1 to T2. SCS-Isolation, brief version of the Self-Compassion Scale—Isolation.

**Figure 3:**
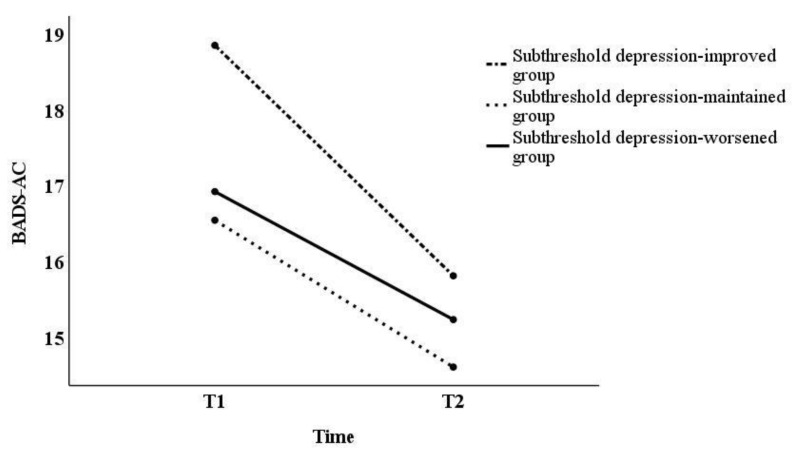
Changes in BADS-AC score from T1 to T2. BADS-AC, Behavioral Activation for Depression Scale—Activation.

Although prior research has pointed out that the normality assumption is a precondition, failure to meet it does not make the results of the analysis significantly different [43]. Hence, the analysis in this study was conducted using two-way repeated measures ANOVAs. In this study, Mauchly’s sphericity test results showed that sphericity was not assumed for all factors (*p* < 0.05). However, if the assumption of sphericity is not satisfied by the Mauchly’s sphericity test, it should be corrected by the Greenhouse–Geisser method [44]. Thus, as sphericity did not hold, the Greenhouse–Geisser correction was used in subsequent tests. Two-way repeated measures ANOVAs yielded significant differences in interaction effects on the BADS-AR [*F*_(2,120)_ = 5.01, *p* < 0.01] and SCS-isolation [*F*_(2,120)_ = 4.96, *p* < 0.01], and the main effect on the BADS-AC [*F*_(2,120)_ = 4.33, *p* < 0.05] (Table 2). Next, we examined the simple main effects of the BADS-AR and SCS-isolation (Figure 1 and Figure 2). For the BADS-AR, a significant difference was observed between the subthreshold depression-improved and the subthreshold depression-worsened groups at T2 (*p* < 0.05). Moreover, the BADS-AR score of the subthreshold depression-worsened group significantly increased one year later (*p* < 0.01) (Figure 1). For SCS-isolation, there was a significant difference between the subthreshold depression-improved and other groups at T2 (subthreshold depression-maintained group [*p* < 0.05], subthreshold depression-worsened group [*p* < 0.01]). The SCS-isolation scores of the subthreshold depression-improved group significantly decreased (*p* < 0.05), while that of the subthreshold depression-worsened group significantly increased one year later (*p* < 0.05). For the main effect, the BADS-AC score significantly decreased from T1 to T2 (Figure 3).

## 4. Discussion

The aim of this study was to examine whether the components of behavioral activation and self-compassion are related to the deterioration of university students from subthreshold depression to MDD. In this study, approximately 30% of university students met the criteria for subthreshold depression before COVID-19, and approximately 11% deteriorated from subthreshold depression before COVID-19 to depression during the COVID-19 pandemic. According to the results, the BADS-AR and SCS-isolation scores significantly increased during the COVID-19 pandemic.

A previous study reported that 24.7% of people had depressive symptoms before the COVID-19 pandemic, while 52.5% had depressive symptoms during COVID-19 [45]. Our results are consistent with those of the previous research [45]; the proportion of university students with depressive symptoms may have increased during the pandemic. A study reported that 11% of the participants with subthreshold depression met the MDD criteria after one year [46]. In comparison, 26.3% of those with subthreshold depression showed the same symptoms, while 62.7% of the participants with subthreshold depression no longer met the criteria [46]. The rate of change to MDD in our study was identical. However, the maintained subthreshold depression rate was higher. Therefore, the abnormal situation of the COVID-19 pandemic might have increased the rate at which university students developed subthreshold depression. In T1, we examined whether there were differences among the three groups regarding the history of treatment at health service centers and hospitals. The results showed no significant differences among the three groups. This suggests that there is no association with the development of MDD in this study. However, we were not able to examine whether the patients received treatment at the health service center or hospital during the one-year observation period. Thus, future studies may need to consider the history of treatment during the observation period. Moreover, there was no significant difference in club or circle activities in the three groups. Although we did not examine the details of club or circle activities, it is possible that the activity restrictions imposed by COVID-19 limited the number of club or circle activities taking place at the university, which could explain the lack of difference among the three groups.

In this study, the BADS-AR scores for the subthreshold depression-worsened group were significantly higher than those in the subthreshold depression-improved group. No significant difference was observed between subthreshold depression-worsened and the subthreshold depression-maintained groups. Previous research has shown a significant difference in the degree of avoidance and rumination between subthreshold depression and depression [47]. The results of this study are inconsistent with those of previous studies. The BADS-AR measures avoidance of a negative aversive state and engagement in rumination, rather than active problem solving [35,36]. A previous study reported that 44% of university students reported experiencing depressive thoughts during the COVID-19 pandemic [1]. Because the COVID-19 pandemic has led to an increase in negative thinking tendencies for many university students, there may not have been a significant difference in the degree of avoidance and rumination between the subthreshold depression-maintenance group and the subthreshold depression-worsening group. Contrastingly, the degree of avoidance and rumination of the subthreshold depression-maintained group did not change significantly from T1 to T2, only the subthreshold depression-worsened group showed a significant increase from T1 to T2. Avoidance behavior is a risk factor for developing MDD [48]. Thus, this result is consistent with those of previous studies [48].

For self-compassion, a significant difference in SCS-isolation was found between the subthreshold depression-improved and other groups. Furthermore, the isolation score of the subthreshold depression-worsened group had significantly increased one year later. Social isolation and loneliness were severe problems during the pandemic [49]. A previous study suggested that loneliness was the leading risk factor for MDD [50] due to the social-distancing policy. Moreover, profoundly lonely participants with the smallest social networks had the highest estimated rates of depression [3]. Therefore, isolation during COVID-19 might have been associated with the pathological change from subthreshold depression to MDD.

No significant differences in the EROS scores between the three groups were detected in this study. The previous study reported no difference in the EROS scores between the subthreshold depression and depression groups, consistent with other research in this area [47]. However, in the present study, the lack of group differences was inconsistent with the previous study [47]. In this study, the BADS-AC scores decreased significantly from T1 to T2. This finding indicates that overall activity decreased from the pre-pandemic to the pandemic period for all groups. In a review article, most studies reported significant decrease in mild physical activity (i.e., walking) among undergraduate students [51]. Moreover, no significant differences were detected in the VQ-Progress scores between the three groups in this study. Regarding behavioral activation mechanisms, activation increased positive reinforcement [14,52]. However, the EROS scores of the subthreshold depression-improved group in this study were lower than those of the healthy group in the previous study [47]. Therefore, during the COVID-19 pandemic, university students’ general activity and frequency of positive reinforcement decreased. From the pre-pandemic to the pandemic period, positive reinforcement frequency in the subthreshold depression-improved group may have been similar to the others.

This study’s results suggest that the frequency of avoidance behavior was higher in subthreshold depression during the COVID-19 pandemic; avoidance behavior is associated with the deterioration from subthreshold depression to MDD. Additionally, the results showed that isolation is related to the onset of MDD. The increased frequency of avoidance behavior may also lead to decreased involvement with others. Consequently, attention to avoidance behavior may be valuable. Therefore, even in extraordinary situations such as the COVID-19 pandemic, behavioral activation, such as modifying avoidance behavior and enhancing feelings of pleasure and accomplishment, may be of value to university students.

This study has five limitations. First, the present study examined the factors associated with pathophysiological deterioration from subthreshold depression to full depression in the context of behavioral activation. Various cognitive and other factors are associated with developing MDD. Future studies should include a wide variety of these factors. Second, only undergraduate students were included in the study sample. Consequently, generalizing these results to the broad population will require the examination of a more extensive and diverse sample. Third, the present study was conducted using PHQ-9 and CES-D to confirm subthreshold depression and depression. Owing to this study being based on a survey conducted online, it did not allow for physician consultations or structured interviews. Future studies might need to be conducted using physician consultations and structured interviews to achieve greater diagnostic accuracy. Fourth, in this study, the number of people in the subthreshold depression-worsened group was small (*n* = 13). Previous studies have suggested that the prevalence of subthreshold depression is 29.2% [6], and that 11% of the participants with subthreshold depression met the MDD criteria after one year [46]. Thus, the rate of change from subthreshold depression to depression may be small. A larger sample of university students may need to be used to examine the factors associated with change in depression status in future studies. Fifth, because this study was conducted with general university students, we did not investigate the students’ majors. It is conceivable that stress levels may differ among different majors. Therefore, future studies of university students might need to examine what they major in. Although this study may have had some limitations, the originality of this study is that the behavioral activation and self-compassion factors revealed that avoidance and isolation are factors associated with the deterioration from subthreshold depression to depression. In addition, the effectiveness of psychotherapy in preventing the deterioration from subthreshold depression to depression has not been demonstrated [12]. It may be that there is a lack of coping with avoidance and isolation revealed in this study. The results of this study may contribute to future treatment as one evidence to consider treatment targets to prevent deterioration from subthreshold depression to depression.

## Figures and Tables

**Table 1:** Results of one-way ANOVA and Fisher’s exact test.

	Subthreshold Depression- Improved Group	Subthreshold Depression- Maintained Group	Subthreshold Depression- Worsened Group		
	*N* = 42 (34.15%)	*N* = 68 (55.28%)	*N* = 13 (10.57%)	*p-*Value	Multiple Comparison
Results of one-way ANOVA					
Mean Age (T2)	21.21 (1.18)	21.57 (1.53)	20.69 (1.38)	0.09	
CES-D (T1)	21.21 (4.82) ^1^	23.79 (5.26) ^2^	26.85 (5.73) ^3^	<0.01	1 < 2, 1 < 3
CES-D (T2)	12.45 (3.62) ^1^	24.18 (5.97) ^2^	32.92 (7.54) ^3^	<0.01	1 < 2 < 3
Results of Fisher’s exact test					
Female/Male	22/20	42/26	6/7	0.46	
Are you a member of any club or circle activities in the university? Yes/No (T1)	27/15	36/32	9/4	0.40	
Have you ever consulted health service center or student counseling service of the university for mental health problems? Yes/No (T1)	1/41	9/59	1/12	0.15	
Have you ever visited a hospital (psychiatry or psychosomatic medicine) for mental health problems? Yes/No (T1)	5/37	7/61	4/9	0.14	

Note: Values in parentheses represent standard deviation. CES-D, Center for Epidemiologic Studies Depression Scale. There is a significant difference between different numbers (1 < 2: *p* < 0.05, 1 < 3: *p* < 0.01, 1 < 2 < 3: *p* < 0.01).

**Table 2:** Results of two-way repeated measures ANOVA.

	Subthreshold Depression- Improved Group	Subthreshold Depression- Maintained Group	Subthreshold Depression- Worsened Group		
	*N* = 42 (34.15%)	*N* = 68 (55.28%)	*N* = 13 (10.57%)		
Measures				F-Value (Time × Group)	*p-*Value
BADS-AC (T1)	18.86 (6.55)	16.54 (6.52)	16.92 (6.21)	0.21	0.81
BADS-AC (T2)	15.81 (7.74)	14.60 (8.57)	15.23 (8.61)		
BADS-AR (T1)	18.57 (7.58)	19.96 (8.35)	17.00 (8.87)	5.01	<0.01
BADS-AR (T2)	18.26 (8.48)	21.19 (7.63)	25.54 (4.54)		
EROS (T1)	24.79 (2.80)	22.16 (3.87)	23.54 (3.97)	1.29	0.28
EROS (T2)	24.76 (2.54)	21.62 (3.64)	21.62 (3.64)		
VQ-Progress (T1)	13.26 (6.03)	11.25 (6.03)	13.15 (4.88)	0.21	0.81
VQ-Progress (T2)	13.74 (5.41)	11.74 (6.33)	12.39 (6.86)		
SCS–Self-kindness (T1)	5.67 (1.52)	5.10 (1.53)	5.54 (2.11)	1.16	0.32
SCS–Self-kindness (T2)	5.60 (1.80)	5.35 (1.57)	5.00 (1.58)		
SCS–Common humanity (T1)	5.79 (1.60)	5.40 (1.81)	5.77 (1.92)	1.82	0.17
SCS–Common humanity (T2)	5.76 (1.76)	6.18 (1.28)	6.39 (1.04)		
SCS–Mindfulness (T1)	6.00 (1.48)	5.84 (1.57)	5.23 (1.48)	0.26	0.77
SCS–Mindfulness (T2)	6.12 (1.60)	6.25 (1.73)	5.69 (1.93)		
SCS–Self-judgment (T1)	5.57 (1.93)	5.96 (2.03)	6.46 (1.85)	1.59	0.21
SCS–Self-judgment (T2)	5.50 (1.71)	6.35 (1.88)	7.62 (1.61)		
SCS–Isolation (T1)	6.36 (1.78)	6.49 (1.75)	6.46 (2.21)	4.96	<0.01
SCS–Isolation (T2)	5.76 (1.85)	6.65 (1.89)	7.62 (1.66)		
SCS–Over-identification (T1)	6.50 (1.88)	6.79 (1.91)	6.85 (1.72)	1.75	0.18
SCS–Over-identification (T2)	6.17 (1.68)	7.07 (1.91)	7.62 (1.76)		

Notes: Values in parentheses represent standard deviation. BADS-AC, Behavioral Activation for Depression Scale—Activation; BADS-AR, Behavioral Activation for Depression Scale—Avoidance/Rumination; EROS, Environmental Reward Observation Scale; VQ-Progress, Valuing Questionnaire—Progress; SCS-Self-kindness, short version of the Self-Compassion Scale—Self-Kindness; SCS-Common humanity, short version of the Self-Compassion Scale—Common humanity; SCS-Mindfulness, short version of the Self-Compassion Scale—Mindfulness; SCS-Self-judgment, short version of the Self-Compassion Scale—Self-judgment; SCS-Isolation, short version of the Self-Compassion Scale—Isolation; SCS-Over-identification, short version of the Self-Compassion Scale—Over-identification.

## Data Availability

The datasets generated for this study will not be made publicly available as the authors do not have permission to share the data.
